# Microtubule Destabilization Is Shared by Genetic and Idiopathic Parkinson’s Disease Patient Fibroblasts

**DOI:** 10.1371/journal.pone.0037467

**Published:** 2012-05-22

**Authors:** Daniele Cartelli, Stefano Goldwurm, Francesca Casagrande, Gianni Pezzoli, Graziella Cappelletti

**Affiliations:** 1 Department of Biology, University of Milan, Milan, Italy; 2 Parkinson Institute, Istituti Clinici di Perfezionamento, Milan, Italy; CNRS, France

## Abstract

Data from both toxin-based and gene-based models suggest that dysfunction of the microtubule system contributes to the pathogenesis of Parkinson’s disease, even if, at present, no evidence of alterations of microtubules *in vivo* or in patients is available. Here we analyze cytoskeleton organization in primary fibroblasts deriving from patients with idiopathic or genetic Parkinson’s disease, focusing on mutations in parkin and leucine-rich repeat kinase 2. Our analyses reveal that genetic and likely idiopathic pathology affects cytoskeletal organization and stability, without any activation of autophagy or apoptosis. All parkinsonian fibroblasts have a reduced microtubule mass, represented by a higher fraction of unpolymerized tubulin in respect to control cells, and display significant changes in microtubule stability-related signaling pathways. Furthermore, we show that the reduction of microtubule mass is so closely related to the alteration of cell morphology and behavior that both pharmacological treatment with microtubule-targeted drugs, and genetic approaches, by transfecting the wild type parkin or leucine-rich repeat kinase 2, restore the proper microtubule stability and are able to rescue cell architecture. Taken together, our results suggest that microtubule destabilization is a point of convergence of genetic and idiopathic forms of parkinsonism and highlight, for the first time, that microtubule dysfunction occurs in patients and not only in experimental models of Parkinson’s disease. Therefore, these data contribute to the knowledge on molecular and cellular events underlying Parkinson’s disease and, revealing that correction of microtubule defects restores control phenotype, may offer a new therapeutic target for the management of the disease.

## Introduction

Parkinson’s disease (PD) is the most common motor neurodegenerative disorder, characterized by the loss of dopaminergic neurons in the substantia nigra. Although it has been extensively studied, its molecular etiopathogenesis is not well understood [1]. Monogenic forms of the disorder account for up to 10% of parkinsonisms, and mutated *parkin* and leucine-rich repeat kinase 2 (*LRRK2*) are responsible for the majority of genetic PD cases [2]. Although parkin and LRRK2 seem to act on different physiological processes, being parkin an E3 ligase catalyzing the addition of ubiquitin to target proteins [3] and LRRK2 a multi-domain protein involved in the regulation of neuronal process elongation [4], their actions converge on microtubules (MTs) [5], [6].

MTs are cytoskeletal polymers built up by α/β tubulin heterodimers, which participate in many cellular functions, such as morphology acquisition, cell migration and intracellular transport. MTs usually show a dynamic behavior switching between slow growth and rapid depolymerization [7]. α-Tubulin post-translational modifications (PTMs) correlate with subsets of MTs that behave differently: tyrosinated (Tyr) MTs are the most dynamic subset, acetylated (Ac) or detyrosinated (deTyr) MTs are more stable pools [8].

Several recent studies have highlighted the crucial role of MTs during PD progression. Indeed, many PD-linked proteins, such as parkin, LRRK2 and α-synuclein, are able to modulate the stability of MTs [9]–[11]. However, nothing has been reported about their ability to regulate α-tubulin PTMs. Further evidence has been obtained from neurotoxic models of PD: both rotenone and 1-methyl-4-phenyl-piridinium (MPP^+^) destabilize MTs *in vitro* [12], [13] and in neuronal cells [14], [15]. Moreover, we have demonstrated the importance of α-tubulin PTMs in PD pathology, reporting that MPP^+^ causes an early change in MT stability [15]. All these data highlight the importance of MT dysfunction in PD experimental models, but the demonstration of MT involvement in human disease is still lacking.

Post-mortem analyses of human brain could reveal molecular alterations present in the very late phases of neurodegenerative diseases, with the great disadvantage of working with dead tissues. On the other hand, peripheral tissues are a unique source of human living cells, and in the last few years they have become reliable models for the identification of molecular alterations and possible therapeutic targets in neurodegenerative disorders, including PD [16]–[19]. As recently highlighted [20], human skin fibroblasts are an easy available and robust PD experimental model, due to some of their peculiarities: they express most of the gene relevant to PD and mirror the polygenic risk factors of specific patient; they comprise the chronological and biological aging other than the environmental exposition, reflecting all the cumulative cell damage of the patient; they make very dynamic contacts as neurons do.

On this basis, here we analyzed fibroblasts from patients with idiopathic PD or from patients carrying mutations in either parkin or LRRK2 to establish whether MT alterations are present in baseline conditions or not. The principal findings we report are the considerable reduction in fibroblast MT mass in PD patients with respect to controls and the rescue resulting from either pharmacological or genetic approaches that stabilize MT system. Thus, our results highlight that MT destabilization occurs in PD patients and it seems to represent a point of convergence of genetic and idiopathic parkinsonisms.

## Materials and Methods

### Ethics Statement and Patients

Primary fibroblasts were obtained by skin biopsies from 25 individuals, whose phenotype and genotype data are summarized in Table 1, and that included 10 healthy volunteers as control group and 15 patients affected by PD, divided into three different pathological groups. Age distribution of each group is reported in the scatter plot (Figure S1), and the statistical analyses did not reveal significant differences in age between control and patient groups. All patients were examined by movement disorder neurologists and clinical diagnosis of PD was established according to the UK Parkinson Disease Society Brain Bank criteria [21], [22]. The *LRRK2* G2019S missense mutation and mutations on the parkin (*PRKN*) gene were screened as previously described [23], [24].

**Table 1:** Phenotype and genotype characterisation of investigated individuals.

	COD	PHENOTYPE	GENOTYPE	SEX	AGE^a^	AGE OF ONSET b
**CONT**	FFF0311991	HEALTHY		F	39	
	FFF0541986	HEALTHY		M	41	
	FFF0191992	HEALTHY		M	43	
	FFF0531978	HEALTHY		F	44	
	FFF0961978	HEALTHY		M	44	
	FFF0401991	HEALTHY		F	46	
	FFF0521978	HEALTHY		M	51	
	FFF0421991	HEALTHY		M	54	
	FFF0422011	HEALTHY		M	69	
	FFF0412011	HEALTHY		F	64	
**PARK**	FFF0302009	AFFECTED	c.C815G (p.C238W); exon 6–7 del.	F	57	30
	FFF1072009	AFFECTED	c.del202_203AG (p.Q34/X43); exon 4–6 del.	M	53	40
	FFF0142009	AFFECTED	c.C924T (p.R275W); exon 3 del.	F	41	22
	FFF0292009	AFFECTED	exon 3 del (homozygotes)	F	69	39
	FFF0902009	AFFECTED	c.del202_203AG (p.Q34/X43) (homozygotes)	M	51	20
	FFF0072010	AFFECTED	c.del202_203AG (p.Q34/X43) (homozygotes)	F	59	39
**LRRK2**	FFF0642009	AFFECTED	p.G2019S (heterozygotes)	F	58	41
	FFF0962009	AFFECTED	p.G2019S (heterozygotes)	M	47	40
	FFF0112010	AFFECTED	p.G2019S (homozygotes)	M	68	63
	FFF0092009	AFFECTED	p.G2019S (heterozygotes)	M	46	35
	FFF0502009	AFFECTED	p.G2019S (heterozygotes)	F	61	46
	FFF0452009	AFFECTED	p.G2019S (heterozygotes)	M	79	72
**PD**	FFF0562009	AFFECTED	X	M	71	66
	FFF0542009	AFFECTED	X	M	68	52
	FFF0202010	AFFECTED	X	M	50	42

a Age at time of skin biopsy and establishment of fibroblast cell line.

b The age at which the patient first noticed a PD-related symptom was considered the age of onset of the disease.

The study was approved by the local ethics committee (Istituti Clinici di Perfezionamento, July 13th 2010) and all participants gave written informed consent.

### Cell Culture and Morphometric Analyses

Human fibroblasts were cultured in RPMI 1640 (Hyclone, Logan, UT, USA) containing 15% foetal bovine serum (HyClone) supplemented with 2 mM L-glutamine, 100 U/ml penicillin, 100 µg/ml streptomycin, at 37°C in a humidified atmosphere, 5% CO_2_. For transfection experiments, human fibroblasts were plated at the density of 5000 cells/well. The day after cells were transiently transfected using Lipofectamine 2000 (Invitrogen) (1∶3 DNA to Lipofectamine ratio, 1.5 µg of DNA per well) and analyzed 24 h after transfection. All the plasmids for parkin silencing and over-expression (Figure S2) were supplied by Dr. Sassone (IRCCS Istituto Auxologico Italiano, Milano, Italy) The plasmids encoding untagged human parkin was generated by in-frame insertion of a PCR-amplified DNA fragment encoding human parkin into the pcDNA4-Myc-HIS vector. The fragment was then subcloned in the pECFP-C1 vector to produce in frame CFP-tagged parkin. As negative control a plasmid encoding EGFP mRNA was used. Plasmid encoding short hairpin RNA (shRNA) selective for human parkin was generated with the Gateway® recombination cloning technology (Invitrogen, Carlsbad, CA). The sequence is: sh-183: 5′ CACCGGATCAGCAGAGCATTGTTCACGAATGAACAATGCTCTGCTGATCC 3′.

The double stranded DNA oligo encoding a sense-loop-antisense sequence to the targeted gene was cloned into the pENTR™/U6 entry vector. The shRNA cassettes was then transferred into the plasmid pBLOCK-iT 3-DEST, suitable for Geneticin® selection. As negative control a plasmid encoding shRNA for bacterial lacZ mRNA was used. LRRK2 constructs [25] were kindly gifted by Dr. Greggio (Department of Biology, University of Padova, Padova, Italy).

For pharmacological treatment, control and patient fibroblasts were plated at the density of 5000 cells/well. The day after cells were incubated 2 h with 10 µM of Paclitaxel dissolved in methanol (Sigma-Aldrich, St Louis, MO), Nocodazole dissolved in methanol (Sigma-Aldrich) or Thiocolchine dissolved in DMSO (provided by Dr. Passarella, Dep. of Industrial and Organic Chemistry, Univ. of Milan, Italy) and then analyzed.

In all assays, the fibroblast passage numbers were matched (<10). For morphometric analyses, 5 to 10 random images per plate were captured using an Axiovert 200 M microscope (Zeiss, Oberkochen, Germany), and analyses were made using digital image processing software (Interactive measurement module, Axiovision, Zeiss). All cells in each image were analyzed.

### Immunofluorescence Microscopy

Cells were fixed with cold methanol or 4% paraformaldehyde and incubated with the following primary antibodies and probes: α-tubulin mouse IgG (clone B-5-1-2, Sigma-Aldrich, St Louis, MO); deTyr tubulin rabbit IgG (Chemicon, Temecula, CA); Tyr tubulin mouse IgG (clone TUB-1A2, Sigma-Aldrich); Ac tubulin mouse IgG (clone 6-11B-1, Sigma-Aldrich); vimentin mouse IgG (clone V6, Sigma-Aldrich); Phalloidin-Tetramethylrhodamine B isothiocyanate and 4′,6-Diamidino-2-phenylindole dihydrochloride (Sigma-Aldrich). As secondary antibodies we used Alexa Fluor™ 568 donkey anti-mouse, and Alexa Fluor™ 488 goat anti-rabbit (Invitrogen, Carlsbad, CA). The coverslips were mounted in Mowiol® (Calbiochem, San Diego, CA)–DABCO (Sigma-Aldrich, St Louis, MO) and examined with the Axiovert 200 M microscope.

### Western Blot Analysis

Whole cell extracts, Triton X-100 soluble and insoluble fractions of human fibroblasts were prepared as previously reported [26]. Equal proportions of each fraction or protein samples from whole cell extracts (25 µg per lane) were separated by 7 or 15% SDS-PAGE and blotted onto PDVF membranes (Immobilon™-P, Millipore, Billerica, MA). Membranes were probed with the following antibodies: α-tubulin mouse IgG (clone B-5-1-2, Sigma-Aldrich, St Louis, MO); β-tubulin mouse IgG (clone Tub 2.1, Sigma-Aldrich); deTyr tubulin rabbit IgG (Chemicon, Temecula, CA); Tyr tubulin mouse IgG (clone TUB-1A2, Sigma-Aldrich); Ac tubulin mouse IgG (clone 6-11B-1, Sigma-Aldrich); microtubule-associated protein 1 light chain 3 rabbit IgG (Sigma-Aldrich); vimentin mouse IgG (clone V6, Sigma-Aldrich); actin mouse IgM (N350, Amersham, Little Chalfont, UK); Caspase 3 rabbit IgG (Enzo Life Sciences Ag., Lausen, Switzerland), GADPH mouse IgG (Biogenesis, Poole, UK); Heat Shock Protein 70 mouse IgG (clone 3A3, Chemicon); Glycogen synthase kinase 3 beta rabbit IgG (Abcam, Cambride, UK); Phospho-Glycogen synthase kinase 3 beta (Ser9) rabbit IgG (Cell Signaling Technology, Beverly, MA); p38 alpha MAP Kinase mouse IgG (clone L53F8, Cell Signaling Technology); Phospho-p38 MAP Kinase (Thr180/Tyr182) rabbit IgG (clone 3D7, Cell Signaling Technology); p44/42 MAPK (Erk1/2) rabbit IgG (clone 137F5, Cell Signaling Technology); Phospho-p44/42 MAPK (Thr202/Tyr204) rabbit IgG (clone D13.14.4E, Cell Signaling Technology); parkin mouse IgG (clone prk8, Sigma-Aldrich). Next, immunoblots were incubated with HRP donkey anti-mouse IgG and HRP goat anti-rabbit IgG (Pierce, Rockfort, IL) or HRP goat anti-mouse IgM (Sigma-Aldrich), and developed using enhanced chemioluminescence (Supersignal West Pico Chemiluminescent, Pierce, Rockford, IL). Immunoblots were scanned with JX-330 color image scanner (Sharp Electronics Europe) and analyzed by ImageJ software (National Institute of Health).

### Statistical Analysis

Statistical analysis was performed using STATISTICA (StatSoft Inc., Tulsa, OK), and significant differences of PD patients versus control fibroblasts, or between groups in rescue experiments, were assessed by one-way ANOVA with Tukey HSD *post hoc* test. Data are expressed as means ± SEM.

## Results

### Morphological Alterations Characterize PD Fibroblasts

**Figure 1:**
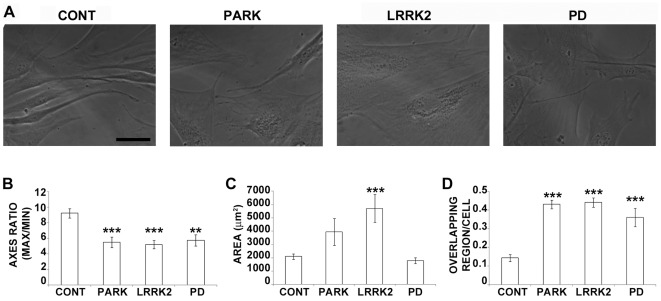
Morphological alterations characterize PD fibroblasts. (A) Representative phase contrast micrographs of cultured human fibroblasts of healthy and PD affected people. Scale bar: 25 µm. Morphometric analysis showed reduced ratio between maximum and minimum axes in parkinsonian fibroblast (B) and increased area in the presence of mutated parkin or LRRK2 (C). (D) Histogram showing the increased number of overlapping regions between cells in patient fibroblasts. *p<0.05 and ***p<0.005 vs control according to ANOVA, Tukey HSD *post hoc* test. All values are expressed as mean ± SEM. CONT = control (N = 10); PARK = patients with mutations of *parkin* (N = 6); LRRK2 = patients carrying mutations in *LRRK2* (N = 6); PD = idiopathic Parkinson’s disease patients (N = 3).

**Figure 2:**
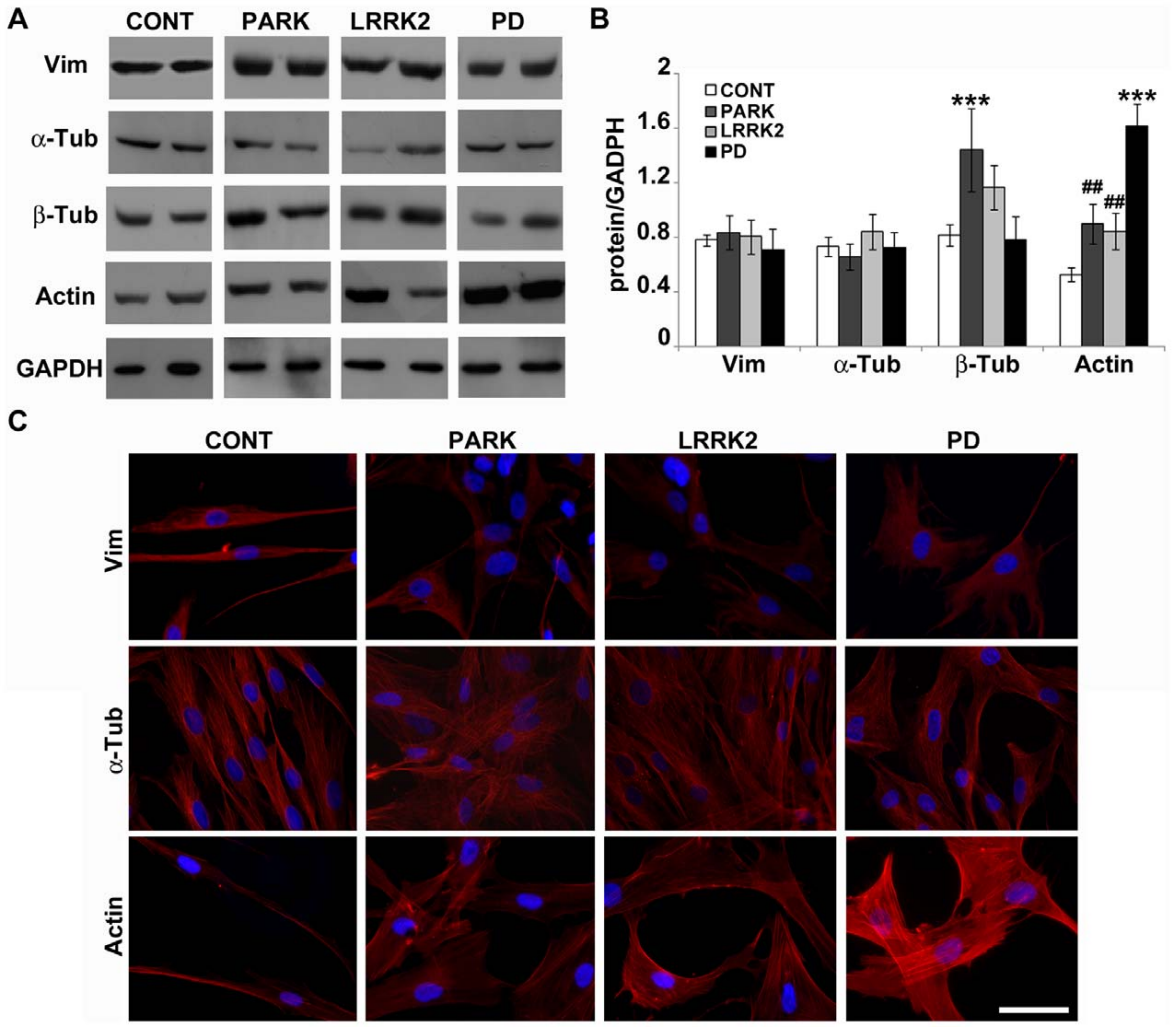
PD fibroblasts show subtle cytoskeleton differences. (A) Immunoblot and (B) densitometric analyses of vimentin (Vim), α-tubulin (α-Tub), β-tubulin (β-Tub) and actin (Actin) were performed in whole cell extracts from human fibroblasts deriving from control (CONT, white bars, N = 10), mutated *parkin* (PARK, dark grey bars, N = 6), mutated *LRRK2* (LRRK2, light grey bars, N = 6) and idiopathic PD (PD, black bars, N = 3). For the quantitation, values of each protein were normalized on the level of GAPDH of the relative sample. All values are expressed as mean ± SEM. *p<0.05 and ***p<0.005 vs control, ##*p*<0.02 vs PD, according to ANOVA, Tukey HSD *post hoc* test. (C) Cultured human fibroblasts were stained with anti-vimentin and anti-α-tubulin primary antibodies or with TRITC-conjugated phalloidin to reveal the organization of intermediate filaments (Vim, top), microtubules (α-Tub, middle) and actin fibers (Actin, bottom), respectively. Concurrent nuclear staining was made by using DAPI (Blue). Scale bar: 20 µm.

We observed striking differences between the cultured human fibroblasts collected from PD patients and those collected from controls in terms of morphology and behavior. Looking at the general morphology of the cells, control fibroblasts were elongated and flanked each other, whereas fibroblasts from PD patients were wider, larger, and partly overlapped, as they lost the ability to sense each other (Figures 1 and 2). Morphometric analyses underlined the decrease of the ratio between maximum and minimum axis (Figure 1B), and the measurement of the area corroborated the idea that PD fibroblasts were larger than control cells (Figure 1C), at least in the presence of mutated parkin or LRRK2. Furthermore, parkinsonian fibroblasts showed a different spatial organization, being much more enshrouded, as pointed out by the increase in overlapping regions (Figure 1D). These data highlight that fibroblasts deriving from PD patients are characterized by altered morphology.

### PD Fibroblasts Show Subtle Cytoskeleton Differences

Since changes in cell morphology are likely mediated by rearrangements of cytoskeletal architecture, we investigated the levels (Figure 2A–B) and organization (Figure 2C) of all the three cytoskeletal polymers: intermediate filaments (IFs), MTs and actin filaments. The levels and localization of vimentin, the principal constituent of the fibroblast IFs, did not vary in PD fibroblasts. Tubulin levels showed changes only in patients carrying parkin mutations, whereas MT organization did not appear to change in any of the experimental groups. It has been reported that parkin promotes α- and β-tubulin degradation and that its PD-linked mutations remove this ability [5]; thus, the enrichment of β-tubulin in patients with parkin mutation is not surprising. On the contrary, α-tubulin levels were unexpectedly unchanged, suggesting possible different regulatory mechanisms that need future and deeper investigations. Finally, we observed a dramatic increase in actin levels in patients with idiopathic PD; phalloidin staining revealed a higher amount of stress fibers that appeared to be randomly oriented whereas in the other experimental groups they were aligned with the major axis of the cells. Thus, these data demonstrate that alterations of the cytoskeleton occur in fibroblasts obtained from patients with parkin mutations and from idiopathic PD patients.

### Impairment of MT Stability is Shared by PD Fibroblasts

**Figure 3:**
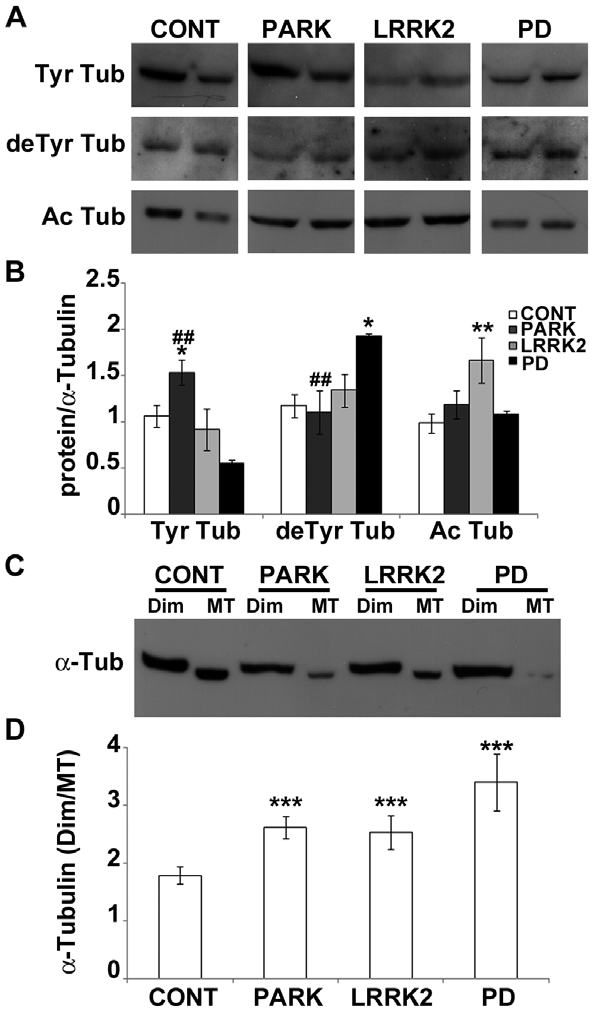
Impairment of MT stability is shared by PD fibroblasts. (A) Immunoblot and (B) densitometric analyses of Tyr, deTyr, and Ac tubulin, were performed in whole cell extracts from human fibroblasts deriving from control (white bars), mutated *parkin* (dark grey bars), mutated *LRRK2* (light grey bars) and idiopathic PD (black bars). For the quantitation, values of each α-tubulin PTM were normalized on the level of α-tubulin of the relative sample. Triton X-100-soluble (free α-tubulin, Dim) and -insoluble fraction (α-tubulin incorporated into MTs, MT) of human fibroblasts were analyzed by (C) immunoblot and (D) densitometric analyses and are shown as ratio. *p<0.05 and ***p<0.005 vs control, ##*p*<0.02 vs PD, according to ANOVA, Tukey HSD *post hoc* test. All values are expressed as mean ± SEM. CONT = control (N = 10); PARK = patients with mutations of *parkin* (N = 6); LRRK2 = patients carrying mutations in *LRRK2* (N = 6); PD = idiopathic Parkinson’s disease patients (N = 3).

Since we have already reported that MT stability plays a crucial role in cultured PC12 cells exposed to MPP^+^ [15], we undertook an in-depth analysis of α-tubulin PTMs and MT mass in human fibroblasts. Western blotting (Figure 3A–B) and immunofluorescence analyses (Figure S3) revealed severe alterations of tubulin PTMs in PD fibroblasts. Parkin mutations induced an increase in Tyr tubulin levels (Figure 3B, dark grey bars), meaning that in the presence of mutated parkin the MT system seemed to be more dynamic. On the other hand, LRRK2 mutation (Figure 3B, light grey bars) caused the enrichment of Ac tubulin, and fibroblasts from patients with idiopathic PD (Figure 3B, black bars) showed a significant increase in deTyr tubulin levels, suggesting that MT (over)stabilization has occurred. The LRRK2-mediated MT stabilization agrees with the results of Gillardon [10], showing that G2019S mutation, the same mutation carried by fibroblasts used here, promotes phosphorylation of β-tubulin and enhances MT stability. We looked further at the α-tubulin PTMs localization (Figure S3). Control cells showed an intense perinuclear Ac tubulin decoration, whereas Ac MTs filled the entire cell body of PD fibroblasts, suggesting that this particular subset of stable MTs had spread, interfering with cell morphology and behavior. Taken together, all these data point out that the alteration of MT stability seems to be a common feature of PD patient fibroblasts. As it has already been reported that PD-inducing neurotoxins affect the state of tubulin polymerization *in vitro* and in neuronal cells [12]–[15], we wondered whether the observed changes in MT stability were correlated with abnormal MT mass in patient fibroblasts. By Western blotting and densitometric analyses (Figure 3C–D) we evaluated the amount of α-tubulin associated with Triton-soluble, i.e. dimeric pool (Dim), and with Triton-insoluble fraction, polymerized MT fraction (MT). The ratio between free α-tubulin versus α-tubulin incorporated into MTs was significantly increased in PD fibroblasts in respect to control cells (Figure 3D), meaning that polymerized MTs were reduced. Thus, our work shows that MT depolymerization is shared by all patient fibroblasts here analyzed and obtained from idiopathic and genetic PD.

### GSK3β Phosphorylation is Reduced in PD Fibroblasts

**Figure 4:**
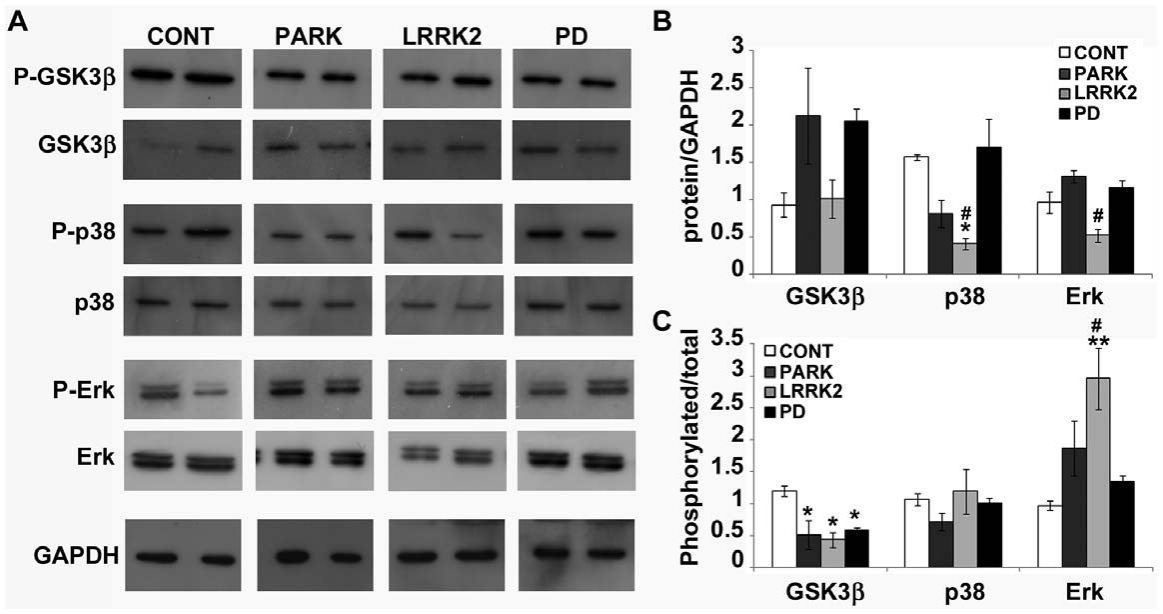
GSK3β phosphorylation is reduced in PD fibroblasts. (A) Immunoblot and densitometric analyses of (B) total and (C) phosphorylated glycogen synthase kinase 3 beta (GSK3β), p38 MAP Kinase (p38) and p44/42 MAPK (Erk) were performed in whole cell extracts from human fibroblasts deriving from control (CONT, white bars, N = 3), mutated *parkin* (PARK, dark grey bars, N = 3), mutated *LRRK2* (LRRK2, light grey bars, N = 3) and idiopathic PD (PD, black bars, N = 3). For the quantitation, values of total protein were normalized on the level of GAPDH of the relative sample, whereas the levels of phosphorylated form were normalized on the values of total protein. All values are expressed as mean ± SEM. *p<0.05 and **p<0.02 vs control, #*p*<0.05 vs PD according to ANOVA, Tukey HSD *post hoc* test.

Looking for a possible explanation for the observed MT destabilization in PD fibroblasts, it is reasonable that Parkin and LRRK2 mutations directly impact MT stability [9], [10]. However, since MT depolymerization is observed also in idiopathic PD fibroblasts, we decided to evaluate the potential implication of signaling pathways converging on MT system. Therefore, in all the PD fibroblast groups, we investigated the level and the activity of glycogen synthase kinase 3 beta (GSK3β), p38 protein (p38) and extracellular signal-related kinases (Erk) that regulate MT stability through the phosphorylation of MT-associated proteins (MAPs). As shown in figure 4, the levels of total GSK3β were highly variable but they did not reach any statistical significance, whereas GSK3β phosphorylation was significantly reduced in all classes of PD fibroblasts (Figure 4C). Total p38 showed a significant reduction only in the presence of mutant LRRK2, whereas phospho-p38 was completely unchanged. On the other hand, LRRK2 induced also a slight decrease of Erk and the significant elevation of phospho-Erk; accordingly to Ren and colleagues [27], fibroblasts from patients with parkin mutation displayed an increase of Erk phosphorylation, although without statistical significance. Nevertheless, phosphorylated GSK3β is the inactive form and the phosphorylation of MAPs by GSK3β promotes their detachment from MT walls [28]. Therefore, showing the significant activation of GSK3β, our data offer a possible mechanistic explanation for the observed MT destabilization in idiopathic PD fibroblasts, but also in cells deriving from patients with genetic cases of the pathology.

### Stress-induced Pathways are not Activated in PD Fibroblasts

**Figure 5:**
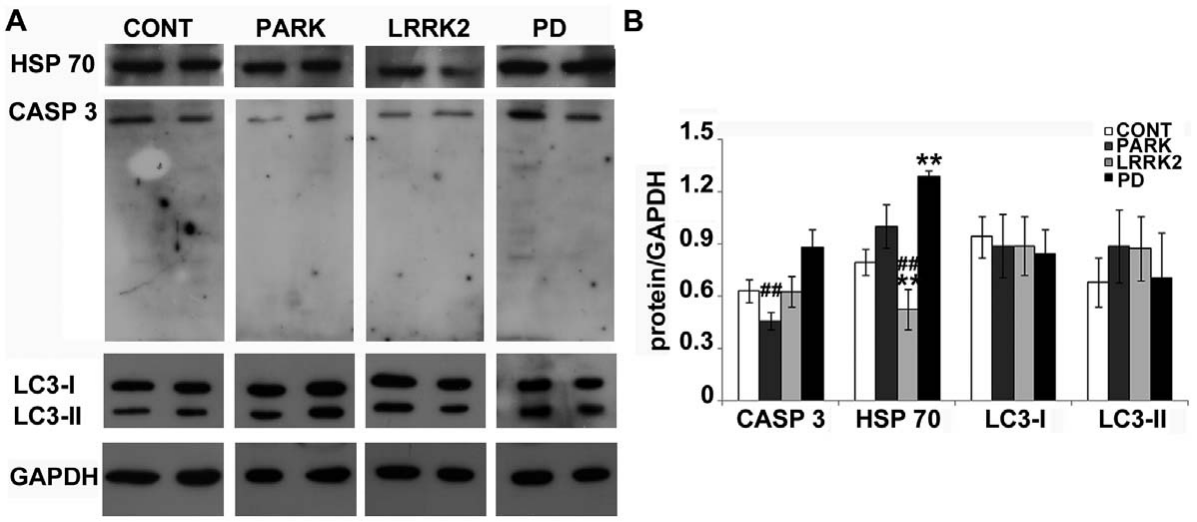
Stress-induced pathways are not activated in PD fibroblasts. (A) Immunoblot and (B) densitometric analyses of caspase 3 (CASP 3), heat shock protein 70 (HSP 70) and microtubule-associated protein 1 light chain 3 (LC3) I and II were performed in whole cell extracts from human fibroblasts deriving from control (CONT, white bars, N = 10), mutated *parkin* (PARK, dark grey bars, N = 6), mutated *LRRK2* (LRRK2, light grey bars, N = 6) and idiopathic PD (PD, black bars, N = 3). For the quantitation, values of each protein were normalized on the level of GAPDH of the relative sample. All values are expressed as mean ± SEM. *p<0.05 and ***p<0.005 vs control, ##*p*<0.02 vs PD according to ANOVA, Tukey HSD *post hoc* test.

MT stability is crucial for the activation of apoptosis and autophagy [29], [30], processes variously related to neurodegeneration in PD [31]. Thus, we decided to analyze the levels of caspase 3 (CASP 3), which is a terminal executioner of apoptosis, and heat shock protein 70 (HSP 70), which is a molecular chaperone with anti-apoptotic properties that prevents aggregation and misfolding of proteins [32]. First of all, the lack of the cleaved form of CASP 3 (Figure 5A) highlighted that there was no activation of the apoptotic programme; moreover, we observed the reduction in the inactive form of the enzyme in the presence of mutated parkin (Figure 5B, dark grey bars). On the other hand, mutant LRRK2 induced a significant reduction in HSP 70 (Figure 5B, light grey bars); interestingly, this finding could explain the higher sensitivity of LRRK2 mutant induced pluripotent stem cell (iPSC)-derived dopaminergic neurons to CASP 3 activation [33]. On the contrary, HSP 70 levels were hugely increased in fibroblasts from idiopathic PD patients (Figure 5B, dark bars). As it has already been reported that HSP 70 prevents MT assembly [34] and stabilizes actin filaments [35], these results, together with the reduction of GSK3β phosphorylation, could easily explain the above reported MT destabilization and the increase in actin filaments in idiopathic PD fibroblasts. Finally, we also looked at microtubule-associated protein 1 light chain 3 (LC3) I and II, well known markers of autophagy. The amount of LC3-II correlates with the extent of autophagosome formation and the conversion of LC3-I to LC3-II is a reliable indicator of autophagic activity [36]. Our results showed no significant changes in the levels of LC3-I and LC3-II (Figure 5), and therefore in PD fibroblasts the autophagic machinery is active at basal level. The levels of LC3-I and -II in LRRK2-linked PD differed from those already reported in knockout mice [37], suggesting that the lack or the mutation of LRRK2 may affect autophagy differently. These data highlight that there is no activation of stress-induced pathways in PD fibroblasts. However, these fibroblasts show MT system alterations that may eventually trigger neuronal death by other mechanisms.

### Pharmacological Stabilization of MTs Rescues Fibroblast Phenotype

**Figure 6:**
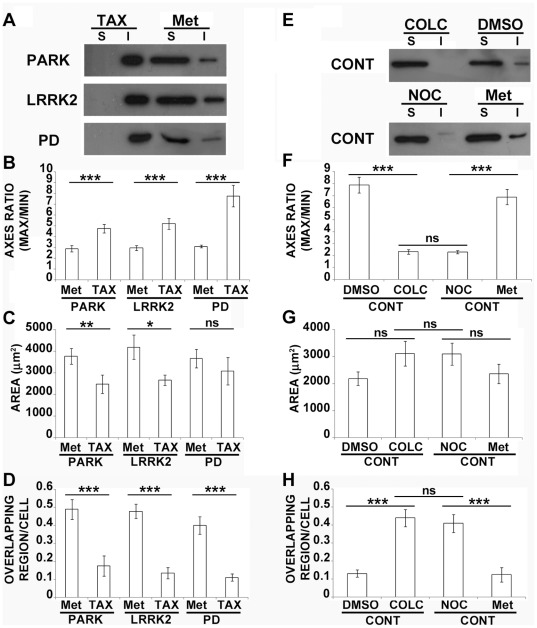
Pharmacological MT stabilization rescues fibroblast phenotype. (A) Representative immunoblot of Triton X-100-soluble (free α-tubulin, S) and -insoluble fraction (α-tubulin incorporated into MTs, I) of patients fibroblasts treated with paclitaxel (Tax) or solvent (Met). Morphometric analyses showing the ratio between maximum and minimum axes (B), the area (C) and the number of overlapping regions between cells (D) of paclitaxel (TAX) or solvent (Met)-treated patient fibroblasts. ns = not significant, *p<0.05, **p<0.02 and ***p<0.005 according to ANOVA, Tukey HSD *post hoc* test. All values are expressed as mean ± SEM. PARK = patients with mutations of *parkin* (N = 4); LRRK2 = patients carrying mutations in *LRRK2* (N = 3); PD = idiopathic Parkinson’s disease patients (N = 3). (E) Representative immunoblot of Triton X-100-soluble (free α-tubulin, S) and -insoluble fraction (α-tubulin incorporated into MTs, I) of control fibroblasts treated with colchicine (COLC), nocodazole (NOC) or solvents (DMSO or Met). Morphometric analyses showing the ratio between maximum and minimum axes (F), the area (G) and the number of overlapping regions between cells (H) of colchine (COLC, N = 5), nocodazole (NOC, N = 5) or solvent (DMSO or Met, N = 5 respectively)-treated control fibroblasts. ns = not significant and ***p<0.005 according to ANOVA, Tukey HSD *post hoc* test. All values are expressed as mean ± SEM.

To validate the idea that MTs and MT destabilization are crucial players in altering cell functions and behaviors in PD conditions, we decided to treat patient derived fibroblasts with taxol (Figure 6), a potent MT stabilizer that has proven to be neuroprotective in midbrain dopaminergic neurons in cultures [14]. After 2 h of treatment with 10 µM of Taxol, tubulin was completely shifted toward the Triton-insoluble fraction (Figure 6A), meaning that there was an increase in the MT pool in patients fibroblasts. The morphometric analyses (Figure 6B–D) showed that the increase in MTs correlated with a correction of cell morphology and behavior, pointed out by the increase of the ratio between maximum and minimum axis and by the reduction of overlapping regions. Furthermore, we treated control fibroblasts with colchicine or nocodazole, two well known MT destabilizing drugs. As expected, we observed almost all the tubulin associated to the unpolymerized pool (Figure 6E), the reduction of the axes ratio and the dramatic increase of overlapped cells (Figure 6F–H), showing that a direct interference with the MT system is sufficient to induce the same alterations we observed in PD fibroblasts (Figure 1). Taken together, these data demonstrate that impairment of MT stability in PD patient derived cells is directly correlated to changes in morphology and behavior, and strongly suggest that MT system may be a good “druggable” candidate for restoring the proper cell mechanics.

### Genetic Manipulation Restores MT Stability and Rescues Fibroblast Phenotype

**Figure 7:**
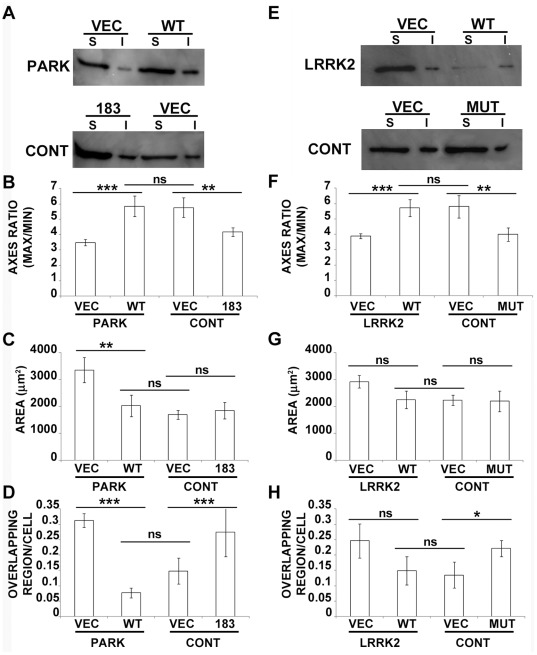
Genetic manipulation restores MT stability and rescues fibroblast phenotype. (A) Representative immunoblot of Triton X-100-soluble (free α-tubulin, S) and -insoluble fraction (α-tubulin incorporated into MTs, I) of fibroblasts collected from patients with parkin mutations (PARK) transfected with control plasmid (VEC) or WT parkin (WT), and of control fibroblasts (CONT) transfected with short hairpin RNA, sh-183 (183) or control shRNA (VEC). (B-D) Morphometric analyses of patients fibroblasts expressing control plasmid (PARK-VEC, N = 4) or WT parkin (PARK-WT, N = 4), and control fibroblasts transfected with control shRNA (CONT-VEC, N = 4) or silenced with sh-183 (CONT-183, N = 4), showing the ratio between maximum and minimum axes (B), the area (C) and the number of overlapping regions between cells (D). ns = not significant, **p<0.02 and ***p<0.005 according to ANOVA, Tukey HSD *post hoc* test. All values are expressed as mean ± SEM. (E) Representative immunoblot of Triton X-100-soluble (free α-tubulin, S) and -insoluble fraction (α-tubulin incorporated into MTs, I) of fibroblasts collected from patients with LRRK2 mutations (LRRK2) transfected with control plasmid (VEC) or WT LRRK2 (WT), and of control fibroblasts (CONT) expressing control plasmid (VEC) or G2019S mutant LRRK2 (MUT). Morphometric analyses of patients fibroblasts expressing control plasmid (LRRK2-VEC, N = 3) or WT LRRK2 (LRRK2-WT, N = 3), or of control fibroblasts transfected with control plasmid (CONT-VEC, N = 3) or G2019S mutant LRRK2 (CONT-MUT, N = 3), showing the ratio between maximum and minimum axes (F), the area (G) and the number of overlapping regions between cells (H). ns = not significant, *p<0.05, **p<0.02 and ***p<0.005 according to ANOVA, Tukey HSD *post hoc* test. All values are expressed as mean ± SEM.

To further consolidate our results, we decided to perform rescue experiments, by over-expressing the wild-type (WT) parkin or LRRK2 in the fibroblasts from patients bearing the mutations in parkin or LRRK2, respectively. Moreover, in order to validate the idea that genetic manipulations of these proteins directly influence MT system, and therefore cell architecture, we tried to affect control fibroblasts either by parkin silencing or by mutant LRRK2 expression. As reported in figure 7A, transfection of WT parkin increased polymerized MTs in patient fibroblasts, whereas parkin silencing reduced MT fraction in control cells. Consistent with our hypothesis, the analyses of cell morphology revealed that expression of WT parkin increased the axes ratio and reduced overlapping regions whereas its silencing exerted the opposite effects (Figure 7B–D), mimicking changes observed in patient fibroblasts. In the same way, over-expression of WT LRRK2 in patient fibroblasts increased MT fraction (Figure 7E), and induced a correction of cell morphology (Figure 7F–H), as highlighted by increased ratio between maximum and minimum axis. Similarly, the expression of mutant LRRK2 in control cells promoted MT destabilization, represented by the increase of free tubulin (Figure 7E), and worsened fibroblast morphology and behavior (Figure 7F–H), as showed by the reduced axes ratio and by the increased overlapping regions. Our data, not only demonstrate that alteration of cell morphology and behavior in genetic PD patient fibroblasts are dependent on impairment of MT stability, but, further, our results make light on the capacity of WT parkin or LRRK2 to correct cell defects by restoring MT stability. This point is further sharpened by the absence of significant differences between the morphology of patient fibroblasts transfected with WT parkin or LRRK2 and the cells from healthy subjects expressing control vectors, indicating that the correction of MT system is sufficient to rescue the cell architecture. Together with the pharmacological experiments, these data reinforce the idea of a pivotal role of MT destabilization, and make concrete the hypothesis of a possible MT-based PD therapy.

## Discussion

In this study, we demonstrate that MT stability is impaired in human fibroblasts derived from genetic PD patients and it is likely compromised in idiopathic PD patients, reporting the alterations of α-tubulin PTMs and the significant MT depletion. It has been already shown that human fibroblasts carrying *parkin* with the deletion of the 4^th^ exon, encoding the MT binding domains, show a higher degree of MT depolymerization when they challenged with a MT disruptor agent such as colchicine [27]. Here we report that MT depolymerization occurs in PD fibroblasts even without the addition of any stressor, and that MT destabilization seems to be a common feature shared by idiopathic and genetic parkinsonism. It is noteworthy, as we demonstrate here, that both pharmacological treatment and genetic approaches are able to restore the proper MT stability and, therefore, to rescue cell alterations deriving from MT destabilization. Thus, our work highlights, for the first time, that MT dysfunction is present in patients in baseline conditions and that correction of MT defects recovers cell phenotype, underlining the central role of MT system in PD.

Tubulin PTMs have recently been linked to neurodegenerative processes [38]. Our results, indeed, reveal the importance of α-tubulin PTM dysregulation in PD etiopathogenesis. Being Tyr tubulin the newly synthesized α-tubulin [8], the parkin-induced enrichment of Tyr tubulin can be viewed as an attempt to produce new MTs, as a consequence of the depolymerizaton of the older MTs. In addition, it is a clear sign of the increase of dynamic MTs. On the other hand, the enrichment of stable MTs, observed in idiopathic PD and in patients carrying mutations of LRRK2, could be the extreme effort of the cell to stabilize a collapsing system. In any case, both the hyper-dynamicity caused by mutant parkin and the over-stabilization associated with LRRK2, actually represent an imbalance of MT dynamics. Thus, the first outcome of our work is the suggestion of a new biological mechanism for LRRK2- and parkin-mediated regulation of MT stability, i.e. the modulation of α-tubulin PTMs.

Neurons are not-dividing cells with an extremely long life, and in their axons accumulate very stable MTs, that remain for much longer time than the usual MT half-life [39]. Thus, the accumulation of aberrant tubulin dimers is likely to occur, especially if tubulin turnover is compromised. This is exactly the scenario we hypothesize for PD patients carrying parkin mutations on the basis that parkin promotes ubiquitination and degradation of tubulin [5] and that β-tubulin significantly increases in the presence of mutated parkin, as we reported in the present work. Moreover, a particularly long life of MTs could lead to an unconventional subset of tubulin PTMs, and the impairment of tubulin PMTs could have further impacts on neuronal functions, being crucial for the regulation of various MT-dependent functions. Through the modulation of binding and velocity of motor proteins, tubulin PTMs are supposed to be involved in the regulation of axonal transport [40] whose impairment has recently been suggested as a common and early event in neurodegeneration [41]. We have previously reported that imbalance of α-tubulin PTMs results in impairment of axonal transport and in mitochondrial damage in PC12 cells exposed to MPP^+^ [15]. Here we show that parkin and LRRK2 modulate tubulin PTMs, offering alternative explanations for the reported capacity of parkin to arrest the movement of damaged mitochondria [42] and for the ability of LRRK2 to modulate trafficking and distribution of synaptic vesicles in cortical neurons [43]. Furthermore, we observe the significant activation of GSK3β in PD fibroblasts, that with its upstream and downstream regulators has key roles in many neuronal processes [28], as neurite outgrowth, neuronal polarization and, perhaps, axonal transport. Active GSK3β phosphorylates MAPs with the consequent MT depolymerization and the breakdown of the railways along which motor proteins move; therefore, an increase in GSK3β activation can likely affect axonal transport. Thus, having shown the ability of parkin and LRRK2 to modulate tubulin PTMs and MT-related signaling pathways, the present paper could be a good starting point to analyze the ability of parkin and LRRK2 to regulate axonal transport.

The proper regulation of MT dynamics is critical for the survival and for the establishment of cell-cell contacts in different cell types [44], [45]. For example, when fibroblasts collide they undergo contact inhibition of locomotion that involves cell retraction and reversal of polarity, allowing cells to change the direction of migration and to move in a cell free environment. During aging, fibroblasts motility declines contributing to deficits in wound-healing, and this impaired behavior has been associated to disorganization of actin cytoskeleton [46]. Further data confirmed that mechanical properties are altered in consequence to the increased amount of polymerized actin in fibroblasts from old donors, whereas no significant changes in vimentin or MTs content are associated with aging process [47]. Very recently, Kadir and colleagues [45] have shown that this behavior resides on the fine tuning of MT dynamics and organization, especially at the sites of cell contact, where MT dynamics shall rise above a threshold to permits contact inhibition of locomotion; they also reported that Y24632-treated cells, which have hyper-stable MTs, are unable to re-orientate. Here, we demonstrate that PD patient fibroblasts have altered morphology and spatial organization that could be explained by the increased of stable MTs in LRRK2 and idiopathic PD, but also by the spreading of Ac MTs in all PD fibroblasts, that would locally interferes with the acceptable MT dynamics. Therefore, our data show that changes in MT stability are specifically associated to PD conditions and suggest that PD pathology could reside on compromised cell mechanics due to a failure of the MT system. This idea is strengthened by the fact that the administration of taxol, a MT stabilizing agent, or the expression of either WT parkin or WT LRRK2 in PD patient fibroblasts, provokes an increase in the polymerized MTs and a recovery of the cell morphology and behavior. Interestingly, the MT destabilization observed in patient fibroblasts unravels a possible intrinsic MT weakness in PD affected people that could be crucial for neuronal survival and especially for dopaminergic neurons, being shown to be particularly vulnerable to the colchicine-induced MT depolymerization [14], [48].

Mitochondrial dysfunction has been related to the pathogenesis of PD for a long time, and recent papers show that both parkin [17] and LRRK2 [18] can be important for the regulation of mitochondria function and malfunction. In the last few years, tubulin has proved to be able to modulate mitochondrial respiration through its interaction with voltage-dependent anion channels, the most abundant protein in the mitochondrial outer membrane. In particular, it has been reported that tubulin decreases the respiration rate of isolated mitochondria [49] and that the increase in tubulin dimers induces mitochondrial depolarization in human cancer cells [50]. Under this light, the increased amount of free tubulin we observed in human PD fibroblasts could be responsible for the mitochondrial alterations in these cells, described elsewhere [17], [18]. Thus, as we and others have already suggested [14], [15], [51], MTs and mitochondria collaborate in producing dopaminergic neuron death in PD.

Taken together, our results highlight, for the first time, that proteins associated with PD, such as parkin and LRRK2, have an impact on MT organization and stability in humans, and that idiopathic PD seems to display MT impairment as well. Furthermore, our analyses reveal that these MT alterations profoundly affects cells morphology and behavior, but also that MT stabilization, by taxol treatment or by expression of WT parkin or WT LRRK2, is sufficient to restore the correct cell mechanics. The groundbreaking technique of iPSC-derived dopaminergic neurons [52] offers the noteworthy advantage of recapitulating key molecular aspects in a human model of neurodegeneration, and, moreover, iPSCs enable the production of patient-specific cell lines, with the potential use for high-throughput drug screening and personalized therapies. We will move onto this exciting field soon to validate in human neurons the occurrence of MT dysfunction and to seek a possible MT-based therapy, trying to transfer to neurons our actual findings in human fibroblasts as well as to deeper investigate the biological relationship among parkin, LRRK2 and MTs. Thus, the present work can be the launch pad for the study of MT system in PD patients.

## Supporting Information

Figure S1 **Age distribution in the experimental groups.** Scatter plot representing the age distribution of the individuals in each experimental group. CONT = control (N = 10); PARK = patients with mutations of parkin (N = 6); LRRK2 = patients carrying mutations in *LRRK2* (N = 6); PD = idiopathic Parkinson’s disease patients (N = 3). Statistical analyses did not reveal differences in age between control or patient groups (*p* = 0.168 according to ANOVA).
(TIF)

Figure S2 **Parkin over-expression and silencing.** (A) Representative micrographs of cultured fibroblasts deriving from PD affected patients bearing parkin mutation transfected with control plasmid (PARK+VEC) or WT parkin (PARK+WT). Scale bar: 20 µm. (B) Representative immunoblot of parkin performed on cultured fibroblasts deriving from healthy subjects transfected with control shRNA (CONT+VEC) or silenced with sh-183 (CONT+183).
(TIF)

Figure S3 **PD fibroblasts show altered α-tubulin PMT staining.** Human fibroblasts were immunostained for Tyr, deTyr and Ac tubulin, to investigate MT organization and stability. All cells were concurrently stained with DAPI (blue), to visualize the nucleus. Scale bar: 25 µm. CONT = control; PD = idiopathic Parkinson’s disease; PARK = patients with mutations of *parkin*; LRRK2 = patients carrying mutations in *LRRK2*.
(TIF)
