# Correction: Microtubule Destabilization Is Shared by Genetic and Idiopathic Parkinson’s Disease Patient Fibroblasts

**DOI:** 10.1371/annotation/6db7193b-913a-42f2-aa7c-139d6e15142a

**Published:** 2012-08-06

**Authors:** Daniele Cartelli, Stefano Goldwurm, Francesca Casagrande, Gianni Pezzoli, Graziella Cappelletti

This correction is issued to include the previous papers by Esteves and colleagues which contribute to discuss the crucial role of microtubule dysfunction in the molecular mechanisms underlying Parkinson's disease but were unfortunately omitted from the list of cited references:

Esteves AR, Arduino DM, Swerdlow RH, Oliveira CR, Cardoso SM (2009) Oxidative stress involvement in α-Synuclein oligomerization in Parkinson's disease cybrids. Antioxid Redox Signal 11: 439-448.

Esteves AR, Arduino DM, Swerdlow RH, Oliveira CR, Cardoso SM (2010) Microtubule depolymerisation potentiates alpha-synuclein oligomerization. Front Aging Neurosci 1: 5.

Dr. Esteves and colleagues used a Parkinson's disease-derived cellular model, as cytoplasmic hybrid (cybrid) cell lines are, in which the mitochondrial DNA obtained by platelets of patients affected by idiopathic Parkinson's disease was transferred to human neuron-committed teratocarcinoma cells, previously depleted of endogenous mitochondrial DNA. Compared to a control cybrid cell line, the Parkinson's disease line showed an increased free/polymerized tubulin. These data are in agreement with our article that highlights as primary fibroblasts obtained by patients affected by genetic or idiopathic Parkinson's disease have a reduced microtubule mass.

Dr. Esteves and colleagues suggested that the alteration of microtubule integrity is dependent on mitochondrial dysfunction, whereas our previous data proposed that microtubule destabilization leads to mitochondria damage (reference 15 in the article). So far, the story is not clear cut; nevertheless, all these data support the idea that microtubules and mitochondria collaborate in producing dopaminergic neuron death in Parkinson's disease. 

