# Supplementary figures and images for: Microtubule Destabilization Is Shared by Genetic and Idiopathic Parkinson’s Disease Patient Fibroblasts
Source: PLoS One. 2012 May 22;7(5):e37467. doi: 10.1371/journal.pone.0037467 (PMC3359730; doi:10.1371/journal.pone.0037467)

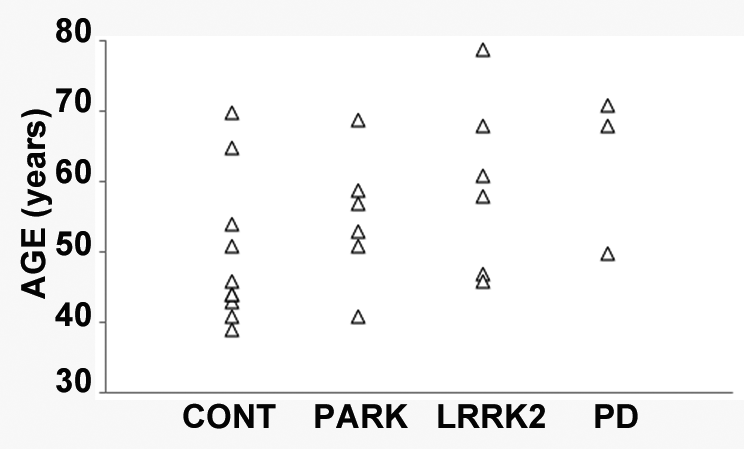

Supplement: Figure S1 — Age distribution in the experimental groups. Scatter plot representing the age distribution of the individuals in each experimental group. CONT = control (N = 10); PARK = patients with mutations of parkin (N = 6); LRRK2 = patients carrying mutations in LRRK2 (N = 6); PD = idiopathic Parkinson’s disease patients (N = 3). Statistical analyses did not reveal differences in age between control or patient groups (p = 0.168 according to ANOVA). (TIF) [file pone.0037467.s001.tif]

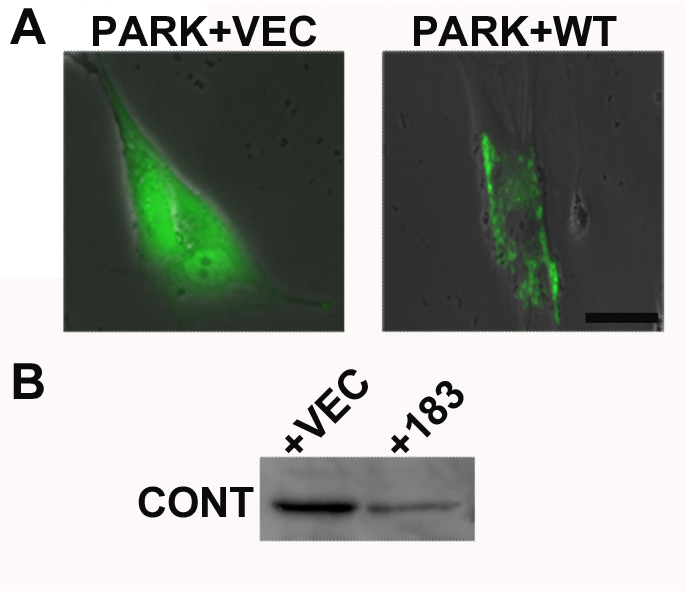

Supplement: Figure S2 — Parkin over-expression and silencing. (A) Representative micrographs of cultured fibroblasts deriving from PD affected patients bearing parkin mutation transfected with control plasmid (PARK+VEC) or WT parkin (PARK+WT). Scale bar: 20 µm. (B) Representative immunoblot of parkin performed on cultured fibroblasts deriving from healthy subjects transfected with control shRNA (CONT+VEC) or silenced with sh-183 (CONT+183). (TIF) [file pone.0037467.s002.tif]

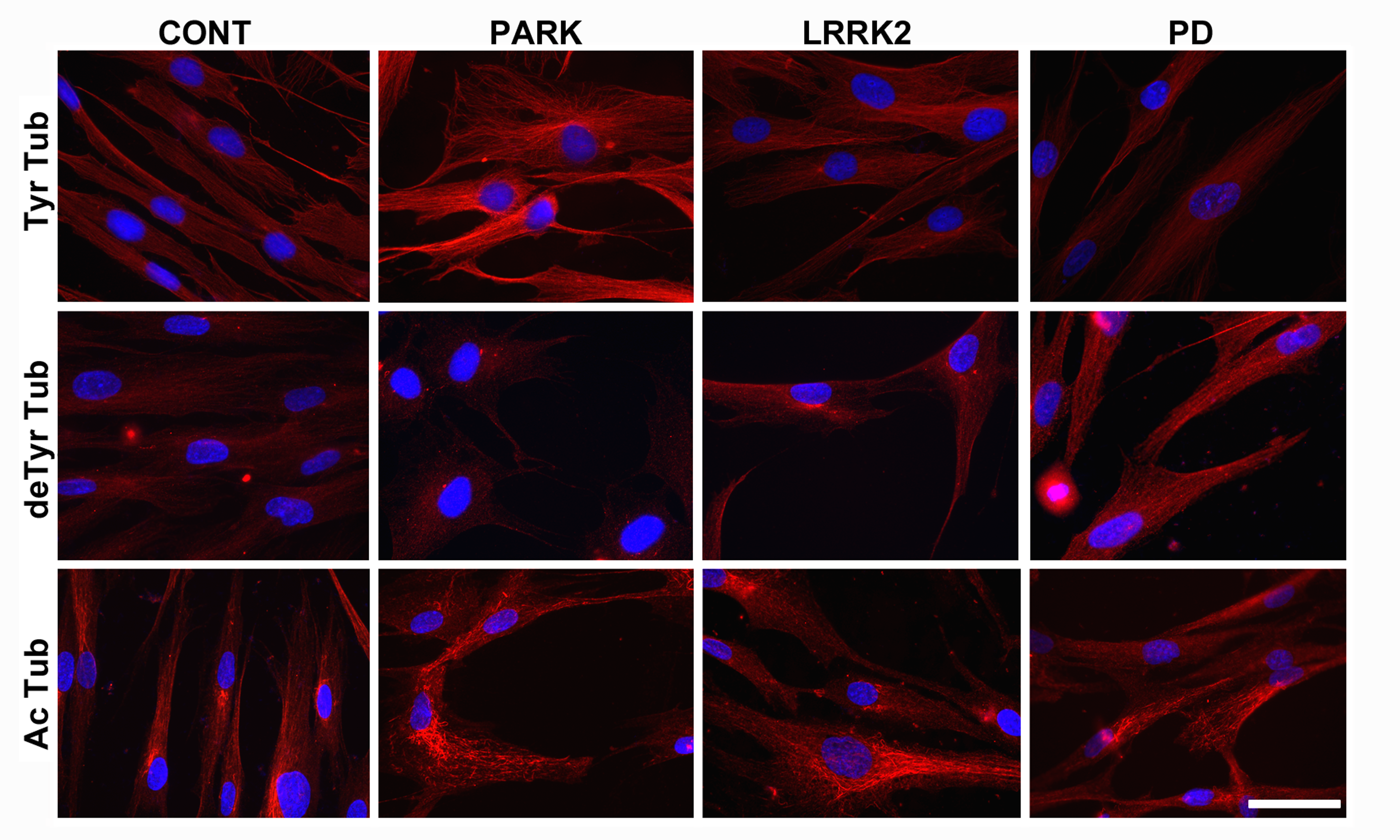

Supplement: Figure S3 — PD fibroblasts show altered α-tubulin PMT staining. Human fibroblasts were immunostained for Tyr, deTyr and Ac tubulin, to investigate MT organization and stability. All cells were concurrently stained with DAPI (blue), to visualize the nucleus. Scale bar: 25 µm. CONT = control; PD = idiopathic Parkinson’s disease; PARK = patients with mutations of parkin; LRRK2 = patients carrying mutations in LRRK2. (TIF) [file pone.0037467.s003.tif]
